# The esthetic rehabilitation of misplaced dental arch after fracture of anterior maxillae: a case report

**DOI:** 10.4076/1757-1626-2-8723

**Published:** 2009-08-25

**Authors:** Suha Turkaslan, Çagri Turna

**Affiliations:** Department of Prosthodontics, Faculty of Dentistry, Suleyman Demirel UniversityIsparta, 32200Turkey

## Abstract

**Introduction:**

In cases where the patient experiences trauma, the teeth may be fractured or a bone segment could be fractured causing misplaced teeth. Ceramic veneer restorations may be a solution for re-establishing the ideal position. The treatment of misplaced dental arch segment is presented and treatment options are discussed.

**Case presentation:**

A 30-years-old female Turkish patient had a history of a dentoalveolar trauma and a surgical operation. Her main complaints were about the unpleasant appearance of her anterior teeth. The maxillary anterior teeth were positioned labially and had irregularities in vertical position inharmonious with each other and the horizontal plane. The misplaced sector treated with minimally invasive dentin bonded porcelain laminate veneers.

**Conclusion:**

Ceramic veneers can be a solution for patients with malpositioned anterior teeth even the situation is severe and excessive tooth reduction is needed.

## Introduction

Dental trauma and injuries due to various causes are not uncommon in society. The most common injuries in the permanent dentition are due to falls, followed by traffic injuries, acts of violence and sports [1]. Maxillary incisors are the teeth with the greatest visibility during common functions and are the most susceptible to fractures by direct trauma, especially in children and teenagers, because of their size and position [1,2]. Injury can lead to displacement, rotation and intrusion of permanent anterior teeth [3].

Dental trauma requires adequate treatment, specific to each fracture in order to preserve the remaining teeth and their position [4]. Reestablishment of proper esthetics and function in the anterior region is quite important for the patient. The relentless pace of innovation and development in restorative materials culminates in offering clinicians a whole plethora of esthetic materials with different techniques. All-ceramic veneers provide precise colour match and translucency to the natural tooth and fulfil the need for adequate retention [5]. Porcelain laminate veneers are very suitable for young adults who have large pulp chambers and pulp horns close to the teeth enamel surface. For these types of restorations, the preparation does not cause the reduction of axial walls. This results in preservation of tooth structure and the surrounding hard and soft tissue architecture [6]. Clinical performance of porcelain laminate veneers is found reliable. A ten year clinical follow up revealed high success rates (92%) for five years and moderately high success rates (64%) for another five years [7]. Similar results have found by other researchers [8].

Fractures involving enamel and dentin can be restored by simple composite or porcelain laminate veneer restorations. Some degree of anterior teeth displacement and rotation can also be rehabilitated using ceramic veneer restorations [2,4,9].

This case report presents an esthetic rehabilitation of anterior teeth due to misplacement. The indication is not a “classic” congenital misplacement but occurred due to malpractice during surgical operation.

## Case presentation

**Figure 1.**
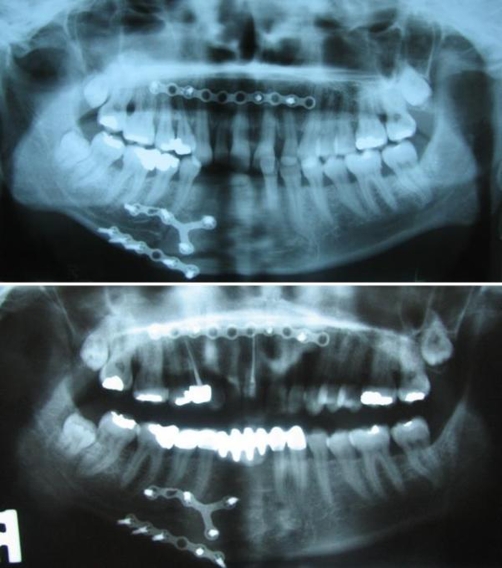
The radiographic examination indicates the superior position of the maxillary anterior segment to the occlusal plane.

**Figure 2.**
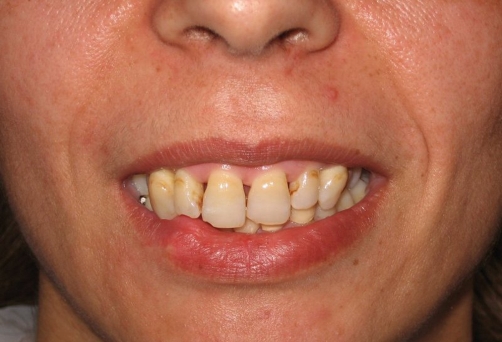
Traumatized and fractured anterior teeth, the right canine and lateral are more inclined than the centrals.

A 30-years-old female Turkish patient referred to our clinic with compliant of unpleasant appearance of her anterior teeth. Patient has a history of a dentoalveolar trauma and a surgical operation before being referred to a dentist. She indicated that her smile has changed following the surgical operation which applied after the traffic accident in the year 2004. After the healing period she directed her complaints to the plastic surgeon but the doctor persuade her that the problem was related with her teeth only. Therefore the patient appealed to our clinic. The clinical and radiographic examinations revealed that all of the maxillary incisors were vestibulary positioned. The maxillary right canine along the maxillary incisors had oblique fractures involving enamel. The maxillary left lateral incisor had not fractured and was intact. The position of maxillary segment between the right premolar and left canine was approximately 3 mm lower than its ideal position (Figure 1). Observing that the inferiority of right canine and lateral incisor is higher than the others, there is a probability that the segment not only placed 3 mm below its ideal position but also placed a few degrees deviation off the vertical axis (Figure 2). The misplacement of the segment is explained to the patient and segmental osteotomy is suggested. The patient rejected the suggestion indicating that she is tired of serial surgical operations, thus the malpractice played a negative effect on her psychology as well.

A conservative treatment was taken into consideration. The indication was porcelain laminate veneers for the maxillary incisors and right canine, all ceramic crown for maxillary right first premolar because of excessive hard tissue loss and a metal-ceramic fixed partial denture for the anterior mandible due to economic reasons.

Before the prosthetic treatment the maxillary right central incisor and first premolar was endodontically treated as they were found to be non vital during the vitality test. During preparation for ceramic veneers, the facial surfaces were reduced by 1.5-2 mm which resulted finishing the preparation on dentin and the incisal edges were reduced by 3-4 mm. All the incisors and canine were prepared with a chamfered finishing line with rounded internal line angles. Smooth margins were created to prevent stress concentration zones. Once the preparation was completed, impressions were made using polyvinylsiloxane impression material (Elite H-D, Zhermack, Germany), and cast in vacuum-mixed Type IV dental die stone (Fujirock, GC Corp, Tokya, Japan) according to the manufacturer recommendations. Stone dies were carefully separated from the impressions and two coats of die spacer (Spacer-Tray, Kerr) were applied 0.5 mm short of the finish line of the preparations. The veneers were waxed up to dies and they were fabricated from lithium disilicate-reinforced glass ceramic material, IPS Empress 2, using the heat press technique according to the manufacturer recommendations. After divestment the veneers were finished and glazed.

**Figure 3.**
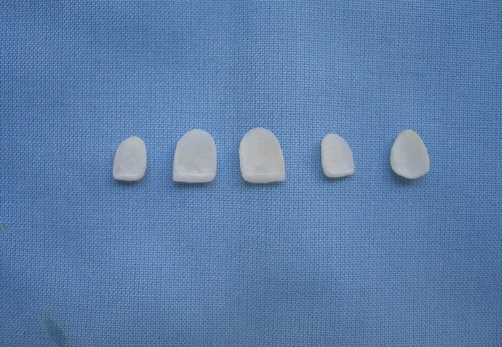
The intaglio surface of porcelain veneers which are ready for cementation.

The inner surface of indirect veneers were treated with air-particle abrasion using 50 μm Al_2_O (Korox, Bego, Germany) with a chairside air-abrasion device (CoJet, 3M-ESPE, Germany) from a distance of 10 mm at a pressure of 250 kPa bar for 10 s. Then each surface treatment was followed by acid etching with 9% hydrofluoric acid (Pulpdent Corporation, USA) prior to silanization (Figure 3). A silane coupling agent (Pulpdent Corporation, USA) was applied to the internal veneer surface for 60 s and air-dried.

During the cementation process each tooth was etched for 15 s using a 37% phosphoric acid etch-gel (Alpha-Etch 37, Dental Technologies, USA). Subsequently, the tooth surface was rinsed thoroughly and air-dried gently. Dentin primer and adhesive were applied as the preparation reached dentin structure, according to the manufacturer instructions (Clearfil, Kuraray). Following the bonding application a thin layer of light polymerizing composite resin luting cement was applied at the intaglio surface of the veneers, placed onto the prepared teeth and light-polymerized for 40 s (Elipar Free Light, 3M ESPE) from palatal, buccal and incisal sides.

**Figure 4.**
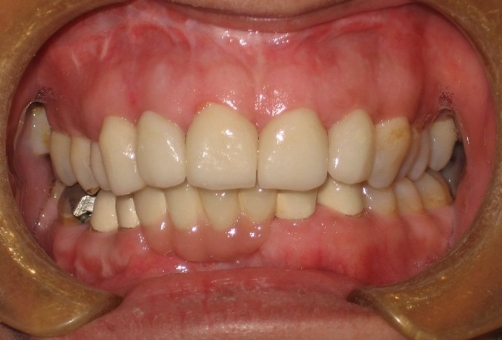
Restorations finished and cemented onto the abutment teeth.

**Figure 5.**
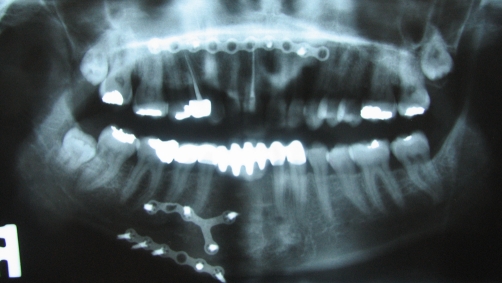
Panoramic radiograph after the restorations cemented.

Excess luting cement was removed and the marginal area was finished and polished with abrasive discs. The metal-ceramic fixed partial denture on the anterior mandibula and the full coverage all ceramic crown for maxillary right first premolar are constructed with conventional procedures and cemented (Figures 4,5). Restorations were checked to avoid any occlusal interference.

**Figure 6.**
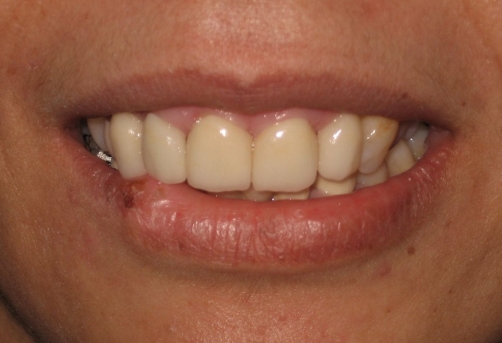
An aesthetic outcome is achieved with restorations in both dental arches. Patient was satisfied with her excellent view of anterior teeth.

**Figure 7.**
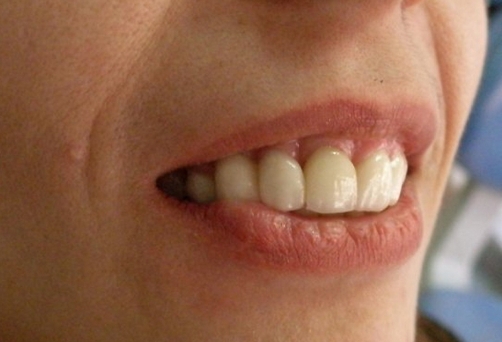
Lateral aspect of the patient. Position, color and form of the teeth are in accordance with the horizontal plane and her smile.

The patient was satisfied with her new smile line and excellent view of the anterior teeth (Figures 6,7). She was recalled in 2 days and encouraged for better dental flossing and also recalled every 6 months for periodical controls. No complication was observed during 3 years clinic service.

## Discussion

The conservative restoration of unaesthetic anterior teeth has been revolutionized by the introduction of laminate veneers. Today, ceramic veneers have become the major modality of treatment when conservative aesthetic restoration of anterior teeth is needed [10,11].

Minimally invasive preparation designs and modern ceramic materials make this treatment option increasingly conservative to the natural tooth structures, while providing both predictable and long-lasting aesthetics. It can be concluded that the bonding systems and procedures used offer reasonable strength and sealing both on the porcelain site and on dentin and enamel [12]. All-ceramic veneers guarantee color and translucency close to those of the natural tooth as well as fulfiling the need for adequate retention, while preserving maximum remaining tooth structure [10,11,13].

Treating large morphological and structural defects demands more than a labial and incisal covering of residual tooth structure. Ceramic veneers involving the incisal edge, proximal areas and larger parts of the palatal surface have been recognized as an alternative to full crowns in the anterior dentition [11,14]. Since full crown preparations require removal of extensive tooth structure, in this case report full coverage restoration of misplaced incisors may cause vitality problems or root canal treatment. Besides the severe reduction in vestibule side, reducing the palatal enamel and some dentin may cause the need of endodontic post applications prior to full crowns.

Today, advancement in dentin adhesives, combined with the highly esthetic resin and ceramic materials in esthetic dentistry give the clinicians a chance to mimic the natural tooth structure. Recently all-ceramic restorations have gained popularity and more frequently preferred in dentistry. The versatility of veneers allows them to be used with a variety of preparation forms, from simple facial veneers to more complex restorations that involve the replacement of more tooth structure [12,14]. Ceramic veneers are one of the most conservative and aesthetic techniques that can be applied when restoring the dental arch for improved aesthetics. The use of ceramic facets to solve esthetic and/or functional problems in the anterior section has been shown to be a convincing option. Years of experience with both the technique and the materials employed offer satisfactory, predictable and lasting results [9,10]. In this case, reducing the anterior teeth to deep dentin layer allowed us to reposition them and correct the vestibule contours while using dentin as bonding surface. Reducing the tooth hard tissues to dentin for porcelain veneer is still a minimally invasive approach to give us an advantage of avoiding any root-canal treatment. It is found that there is a higher risk of failure of veneers on non-vital teeth compared to vital teeth [15].

Porcelain veneers restore the mechanical behavior and microstructure of the intact tooth in vitro even when they are bonded to an extensive dentin surface using an optimized application mode of dentine adhesives [12]. In the current case report the serious reduction was obligatory in order to draw the teeth to their ideal position. Reliable dentin bonding systems enabled the rehabilitation of the case with dentin bonded ceramic veneers.

## Conclusion

Ceramic veneers can be a solution for patients with malpositioned anterior teeth even the situation is severe and excessive tooth reduction is needed.
